# Effects of sorghum rice and black rice on genes associated with cholesterol metabolism in hypercholesterolemic mice liver and intestine

**DOI:** 10.1002/fsn3.1986

**Published:** 2020-11-14

**Authors:** Haiying Liu, Lu Huang, Xinli Pei

**Affiliations:** ^1^ State Key Laboratory of Food Science and Technology Jiangnan University Wuxi China; ^2^ School of Food Science and Technology Jiangnan University Wuxi China

**Keywords:** black rice, cholesterol, hypercholesterolemia, intestinal microbiota, sorghum rice

## Abstract

The effects of different proportions of dietary sorghum rice and black rice on the expression of genes related to cholesterol metabolism in mice liver, intestine, and the characteristics of the small intestinal microbiota were investigated. Six types of diets were used to feed C57BL/6 mice: AIN‐93M standard diet, high‐cholesterol model diet, high‐cholesterol and low‐dose sorghum grain or black rice diet, and high‐cholesterol and high‐dose sorghum grain or black rice diet. The results showed that black rice or sorghum grain diets had no effect on the serum TC, LDL‐C levels in the hypercholesterolemic mice, whereas these diets decreased serum TG level, and black rice diets increased serum HDL‐C level. The diets containing black rice and sorghum grain had no effect on liver TC, TG, HDL‐C levels. However, these diets decreased LDL‐C levels significantly except high dose of black rice. The black rice or sorghum grain diets reduced the expression of the genes encoding liver 3‐hydroxyl‐3‐methyl‐glutarate monoacyl coenzyme A reductase (HMG‐CoA‐R) and increased the expression of SREBP‐2, thereby partially inhibiting the synthesis of cholesterol in liver. The diets containing different proportions of black rice and a low proportion of sorghum grain reduced the expression level of Niemann–Pick type C 1 like 1 (NPC1L1) mRNA and increased the mRNA level of the ATP‐binding cassette transporters, ABCG5/ABCG8, in the small intestine, thereby reducing cholesterol absorption. A diet containing a low proportion of black rice promoted the expression of ABCA1 mRNA and increased the expression of high‐density lipoprotein (HDL) mRNA, thereby promoting reverse cholesterol transport. Black rice diets significantly increased the relative abundances of microbiota in the small intestine and maintained biodiversity, while sorghum grain had no positive effect on the abundance of microbiota.

## Graphical abstract

Some proportions of black rice or sorghum rice are good for serum HDL‐C levels and decreased LDL‐C liver level. Black rice or sorghum rice diets can inhibit cholesterol synthesis in liver and promoted RCT intestine. High dose of black rice increased the relative abundance of intestinal microbiota.

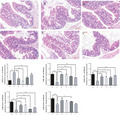

## 1 INTRODUCTION

Cardiovascular disease is the leading cause of death in China. Abnormal cholesterol metabolism in the body leads directly to atherosclerosis, which is the main cause of cardiovascular disease (Kianoush et al., 2016). Ezetimibe is a common drug that acts on the RCT pathway in the intestines to achieve cholesterol reduction, but it has no effect on cholesterol synthesis. Statin drugs are the most common and effective drugs for the treatment and prevention of hypercholesterolemia, but they have certain toxicity, such as increased gastrointestinal discomfort, elevated blood sugar, increased risk of cataract, liver damage, and myopathy (Hu et al., 2012). Therefore, choosing long‐term food therapy with low side effects is one of the most effective methods for lowering cholesterol.

The cholesterol required by the body is mainly synthesized in the liver, and 3‐hydroxyl‐3‐methyl‐glutarate monoacyl coenzyme A reductase (HMG‐CoA‐R) is the main rate‐limiting enzyme in cholesterol synthesis (Reihner et al., 1990). Sterol regulatory element‐binding proteins (SREBPs) are major transcription factors in lipid metabolism, and SREBP‐2 mainly regulates the genes involved in intracellular cholesterol metabolism via the INSIG‐SREBP‐SCAP pathway (Joseph et al., 2002). The excretion of cholesterol mainly depends on bile acids. Liver X receptor (LXR‐ɑ) regulates DNA transcription factors and regulates cholesterol and lipid metabolism in the body via SREBP‐2 and ATP‐binding cassette subfamily A1 (ABCA1) (Wang et al., 2001), respectively. LXR‐ɑ regulates the expression of the cholesterol 7‐alpha hydroxylase gene (*Cyp7a1*) (Mitchell et al., 1991; Pullinger et al., 2002), thus inhibiting bile acid absorption and increasing cholesterol excretion. They are involved in the hepatic cholesterol metabolism and stimulate the excretion of cholesterol into the bile. Therefore, studying biochemical indices and genetic changes of hepatocyte cholesterol metabolism is important in the prevention and treatment of hypercholesterolemia.

Most organs, except for the liver, lack the enzymes that break down steroid hormones. Therefore, cholesterol cannot be excreted by forming bile acids in these organs. High‐density lipoprotein (HDL) is a cholesterol carrier, which transports cholesterol back to the liver for degradation. This process is called reverse cholesterol transport (RCT). The RCT pathway is regulated by a variety of genes, such as the ATP‐binding cassette transporter A and G family (ABCA1, ABCG5/8) (Jia et al., 2011), which are responsible for the binding of free cholesterol and apolipoprotein to form HDL. When the cholesterol level in the body is high, cholesterol is decomposed to form bile acids, which enter the intestine with bile. After bile acids enter the intestine, some are absorbed by the mucosa and return to the liver, the unabsorbed bile acids are degraded to fecal sterols by intestinal microorganisms and excreted (Anderson et al., 2019). The undecomposed cholesterol passes through the surface of the small intestine where it is reabsorbed by the Niemann–Pick C1 Like 1 (NPC1L1) and returns to the liver. This process is the classic enterohepatic circulation (Ahmed & Byrne, 2010). Therefore, the intestine is another important organ for cholesterol metabolism.

In addition, the intestinal microbiota is an important part of the human ecosystem and also associated with cholesterol absorption. A high‐fat diet not only raises cholesterol levels but also severely and rapidly changes the structure of intestinal microbiota. Cani et al. (2007) found that a high‐fat diet causes damage to intestinal permeability and eventually allows harmful bacteria to enter in large quantities. Diets can affect the distribution of intestine microbiota, which in turn seriously affect human health (Possemiers et al., 2009). More and more researchers have begun to focus on the effects of diet on the structure of intestinal microbiota.

In recent years, more attentions have been paid to the functional researches of grains due to their large daily consumptions. Sorghum rice, obtained after the decortication of sorghum seeds, is used as both a medicine and a food in China. However, sorghum rice has drawbacks, such as difficult digestion, astringent taste, which limits its edible value. In addition, the digestibility of sorghum protein is the lowest in cereal crops. Nonetheless, because of the high tannin content in sorghum, it has a certain physiological function. Kim et al. (2015) found that sorghum extract significantly reduced serum triglyceride and total cholesterol in a hypercholesterolemic mouse model and also significantly promoted the expression of CYP7A1 protein in mice. Park et al. (2012) found that sorghum extract significantly increased adiponectin expression in mice, reduced blood glucose levels, and reduced the expression of antitumor necrosis factor in serum.

Black rice refers to coarse black rice with husks removed. It is mainly distributed in Yunnan, Guizhou, and Guangxi, China. Black rice is rich in various trace elements which are at significantly higher levels than that in polished rice, and its nutrient use efficiency is also high. A variety of unique biologically active substances, such as anthocyanin flavonoids, cardiac glycosides, and alkaloids, which are mainly concentrated in the seed coat, confer black rice with strong free radical scavenging effects, cardiovascular protection, and sedation. Hou et al. (2013) found that black rice bran extracts significantly protected liver cells from damage and experiments in vitro showed that anthocyanin was the main active component in liver protection. Watanabe (2016) found that anthocyanin extract promoted mouse bile acid metabolism, improved lipid metabolism, and inhibited oxidative stress in the body.

According to modern Chinese dietary patterns, sorghum and black rice containing foods are only occasionally consumed and are generally not staple foods. As the consumption is low, it is associated with limited health benefits. In order to promote the applications of sorghum and black rice, it is necessary to study different proportions of sorghum and black rice in the diet and their consumption. We hypothesized that different doses of sorghum and black rice may produce different effects on cholesterol metabolism and intestinal microbiota. Therefore, in this study, the effects of black rice and sorghum rice on hepatic cholesterol metabolism, intestinal microbiota, and cholesterol absorption and transport in the intestinal tract of hypercholesterolemic mice were then investigated to provide a basis for dietary treatment of hypercholesterolemia. The molecular mechanisms of these effects were analyzed and the suitable proportions of black rice or sorghum rice in the diet were investigated, to provide useful information on the application of black rice and sorghum rice.

## 2 MATERIALS AND METHODS

### 2.1 Design of animal experiments

Eight‐week‐old male C57BL/6 mice (specific‐pathogen‐free grade, 20 ± 2 g, *n* = 60) were purchased from the Zhaoyan New Drug Research Center Co., Ltd (Suzhou, China) (Animal certificate number: SCXK (SU) 2013–0003). Animal experiments were carried out in the animal experiment center of Jiangnan University (Wuxi, China). All the animal experiments involved had been reviewed and approved by the ethics committee of experimental animals of Jiangnan University (ethical review number: JN. No 20170704–20171015). All animal experiments were carried out complying with the ARRIVE guidelines, the U.K. Animals (Scientific Procedures) Act, 1986 and EU Directive 2010/63/EU for animal experiments.

The animals were maintained on a 12‐hr light/12‐hr dark cycle in an environmentally controlled room (temperature 23 ± 2°C and humidity 60 ± 10%). After 1 week of acclimatization, they were randomly divided into six groups (*n* = 10): group *N* (normal group, fed an AIN‐93M diet), group H (high‐cholesterol group, given a high‐cholesterol diet), group B (low‐dose black rice group, black rice replaced half the maltodextrin and corn starch in the high‐cholesterol diet), group C (high‐dose black rice group, black rice replaced all the maltodextrin and corn starch in the high‐cholesterol diet), S (low‐dose sorghum group, sorghum replaced half the maltodextrin and corn starch in the high‐cholesterol diet), and T (high‐dose sorghum group, sorghum replaced all the maltodextrin and corn starch in the high‐cholesterol diet). The grain compositions were determined in advance, while the change in protein content was regulated by casein and the change in the fat in the feed was regulated by soybean oil. All feeds were prepared by Trophic Animal Feed High‐Tech Co., Ltd, (Jiangsu China). The mice were fed for 12 weeks. The dietary compositions in each group are listed in Table 1. The black rice and sorghum used in the experiment were purchased from Shandong Helaixiang Food Co., LTD. Black rice was from Jingzhou City, Hubei Province, China, and sorghum was from Jinan City, Shandong Province, China.

**Table 1:** Composition of the diets fed to mice

Ingredient^a^ (g/kg diet)	*N*	H	B	C	S	T
Sample^b^	‐	‐	320.80	631.65	284.80	579.65
Cornstarch	465.69	379.35	189.67	‐	189.67	‐
Casein	140.00	114.04	65.04	15.00	88.04	56.00
Dextrinized cornstarch	155.00	126.26	63.13	‐	63.13	‐
Sucrose	100.00	81.46	81.46	81.46	81.46	81.46
Soybean oil	40.00	32.00	13.00	5.00	26.00	16.00
Fiber	50	50	50	50	50	50
Mineral mix	35	35	35	35	35	35
Vitamin mix	10	10	10	10	10	10
L‐cystine	1.8	1.8	1.8	1.8	1.8	1.8
Choline bitartrate	2.5	2.5	2.5	2.5	2.5	2.5
TBHQ	0.008	0.008	0.008	0.008	0.008	0.008
Lard	‐	100	100	100	100	100
Yolk	‐	50	50	50	50	50
Cholesterol	‐	15	15	15	15	15
Bile sodium	‐	2	2	2	2	2
Total weight	1,000	1,000	1,000	1,000	1,000	1,000
Calories (kcal/g)	3.52	3.93	3.87	3.89	3.84	3.75
Lipids(%)	10%	34%	34%	35%	34%	34%
Proteins(%)	17%	13%	13%	13%	13%	13%
Carbohydrates(%)	73%	53%	53%	52%	53%	53%

### 2.2 Collection of samples

After 12 weeks of treatment, the mice were fasted for 12 hr overnight. They were anesthetized with ethyl ether, and blood was collected from their eyes. Their livers were removed, washed with phosphate‐buffered saline (PBS), wiped dry, and weighed. Approximately 0.1 g of fresh liver samples was fixed in 10% formalin neutral buffer for H&E staining. Other livers were stored at −80°C until analysis.

The intact mouse small intestines were taken out. The contents of the small intestine were completely removed with sanitized tweezers in clean bench. Because the intestinal content of each mouse was not enough after fasting, the intestinal contents of three mice were mixed into one sample and placed in cryotubes. The tubes were immediately placed in liquid nitrogen and then stored in a freezer at − 80°C for analysis of microbiota diversity in the mouse intestinal feces. A section of the small intestine near the cecum was taken and fixed in formalin solution for H&E staining and observation. In addition, a section of small intestine was placed in 1 ml of Trizol reagent to determine the expression levels of genes related to cholesterol metabolism in the small intestine.

### 2.3 Biochemical factors in sera and liver homogenates

The blood samples were allowed to stand and were then centrifuged at 2000 rpm for 15 min at 4°C to isolate the sera. Total cholesterol (TC), triglyceride (TG), HDL‐cholesterol (HDL‐C), LDL‐cholesterol (LDL‐C) were examined using commercial kits (Nanjing Jiancheng Bioengineering Research Institute, Nanjing, Jiangsu Province, China) and an Automatic Biochemical Analyzer (Mindray).

About 0.3 g of liver was washed with precooled physiological saline, cleaned, wiped dry, weighed, and homogenized in nine volumes of physiological saline. The homogenate was then centrifuged at 3,000 rpm for 10 min to obtain the supernatant. TC, TG, LDL‐C, HDL‐C were measured with the same method as the blood samples.

Fresh livers and intestines were harvested, fixed, stained with hematoxylin and eosin (H&E), and observed under an XSP‐13CC optical microscope (Caikang Optical Instrument Co., Ltd., Shanghai, China).

### 2.4 RNA extraction and gene expression analysis

The total RNA in the livers and intestines was extracted with an RNA extraction kit (Nanjing Jiancheng Bioengineering Institute, Nanjing, China), according to the manufacturer's protocol, and the cDNA was synthesized with the RevertAid First Strand cDNA Synthesis Kit (TaKaRa Bio Inc.), according to the manufacturer's protocol. The cDNA (1 μl) was diluted (1:10) and used in each Quantitative Real‐time PCR using SYBR Green Supermix (Bio‐Rad) with an Mx3000P instrument (Stratagene). The cycle conditions: predenaturation at 94°C for 5 min, followed by 35 cycles of denaturation at 94°C for 30 s, annealing at 60°C for 30 s, and extension at 60°C for 30 s. The ΔΔCt method was used to quantify the relative gene expression. Differences in mRNA expression were calculated after normalization to 18S rRNA expression. The sequences of the primers used in this study are shown in Table 2.

**Table 2:** Real‐time quantitative PCR primer list

Gene name	Bidirectional primer sequences (5´–3´)	Annealing temperature (°C)	Product length (bp)
Gapdh	F: 5' ATCACTGCCACCCAGAAG 3' R: 5' CTCGTGGAGACGCTTTAC 3'	60	191
Hmgcr	F: 5’ CTTGTTCACGCTCATAGTC 3’ R: 5' GGCTAAACTCAGGGTAATC 3'	60	192
Cyp7a1	F: 5' CGATACACTCTCCACCTTTG 3' R: 5' GGCTGCTTTCATTGCTTC 3'	60	142
Srebf2	F: 5' TGAGGATGAAGGCAAGAC 3' R: 5' AGAACCAGAGGCAGAAAG 3'	60	247
LXR‐ɑ	F: 5' AAGGGAGCACGCTATGTC 3' R: 5' GCTTGAGCCTGTTCCTCTTC 3'	60	185
LDL‐R	F: 5' CCAAGCCTACATAGTGAG 3' R: 5' CCCAGGAATCCAAGTAAG 3'	60	150
SOD1	F: 5' TCCGTCGGCTTCTCGTCTTG 3' R: 5' GTTCACCGCTTGCCTTCTGC 3'	60	154
Nrf2	F: 5' TTTCAGCAGCATCCTCTC 3' R: 5' GGTCACAGCCTTCAATAGTC 3'	60	200
HO‐1	F: 5' CTGGTGATGGCTTCCTTG 3' R: 5' CTCGTGGAGACGCTTTAC 3'	60	216
NPC1L1	F: 5' GGATTCCTACCTGATAGACTAC 3' R: 5' CAGCAATAGCCACATAAGAC 3'	60	212
ABCG5	F: 5' ATTGCCATCCTGACTTAC 3' R: 5' TTTCTATTTCCCGCTCTC 3'	60	166
ABCG8	F: 5' TTGGACAACCTGTGGATAG 3' R: 5' ATAGAGTGGATGCGAGTTC 3'	60	186
ABCA1	F: 5' ACCCACCCTACGAACAAC 3' R: 5' TCTGAGAACAGGCGAGAC 3'	60	187
SR‐B1	F: 5' GAACAGAGCGGAGCAATGG 3' R: 5' CGCAGTTGGCAGATGATGG 3'	60	146

The bacterial DNA in the intestinal feces were extracted using the same method as liver and intestine DNA. The quantities of extracted DNA were measured, the V3 and V4 region flanked by the evolutionary conserved regions were used to design polymerase chain reaction (PCR) primer, and they were then amplified using PCR with the primers. The bacterial 16S rRNA universal prime sequences synthesized and the identified 16S rRNA sequences were analyzed at GENEWIZ Biotechnology Co., Ltd. The DNA library was then used on the Illumina MiSeq platform.

### 2.5 Statistical analysis

The data are presented as means ± standard deviations (*SD*) and were analyzed with GraphPad Prism 6.0 (GraphPad Software, Inc.). Student's *t* test was used to detect differences between group H and group N, B, C, S, T, respectively. Statistical significance was set at *p* < .05 and *p* < .01.

## 3 RESULTS AND DISCUSSION

### 3.1 Effects of black rice and sorghum rice intake on bodyweight and liver weight

**Figure 1:**
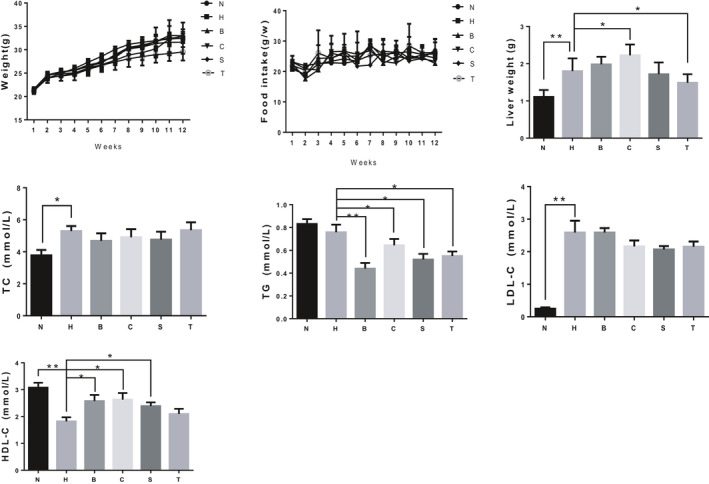
Changes in body weight, food intake during breeding and comparison of liver weight and blood lipids of mice in different groups after breeding. The data are presented as means ± *SD*. Means with *, ** were significantly different (*p* < .05, *p* < .01) compared with group H, without * means no difference (*p* > .05). N: normal group; H: high cholesterol diet group; B: low dose black rice group; C: high dose black rice group; S: low dose sorghum rice group; T: high dose sorghum rice group

In this study, there were no significant differences in body weight and food intake of the high‐cholesterol diet between the groups (Figure 1), suggesting that hypercholesterolemic diet or black rice and sorghum rice treatment had no effects on the growth of mice. Moreover, the mice fed a hypercholesterolemic diet had heavier livers than N and T groups. This suggested that eating sorghum rice can reduce liver weight, but eating excessive black rice can increase liver weight.

### 3.2 Effects of black rice and sorghum rice diets on blood lipids in mice

The results of the blood lipid analysis showed that the serum TC levels in the H were significantly higher than those in *N* (Figure 1), indicating that the mouse model of hypercholesterolemia was successfully established (Matsuno et al., 2001). Diets containing different proportions of sorghum and black rice did not lower the serum TC levels in the hypercholesterolemic mice after the mice were fed the diets for 12 weeks. The trend of serum LDL‐C levels in different groups is similar to that of serum TC levels. There was no significant difference in the serum TG levels between the group H and N, which differed from the serum TC results. However, the diets containing different proportions of sorghum and black rice significantly reduced the serum TG levels in the mice. The serum HDL‐C levels in the group H were significantly lower than those in the group N, but the diets containing Sorghum rice did not change the serum HDL‐C levels in the hypercholesterolemic mice, whereas the serum HDL‐C levels in the group B and C were significantly increased. Black rice and sorghum rice stimulated the body to produce HDL‐C. TC entered the bloodstream; it was then absorbed by HDL‐C and transported back to the liver. This should be the reason for the higher serum TC and HDL‐C levels in each grain group.

Kim et al. (2006) demonstrated that high‐purity anthocyanins had strong antioxidant effects, and the high‐purity anthocyanins extracted from black rice reduced the TC, LDL‐C, and TG levels in the sera of hypercholesterolemic mice (Ho et al., 2012). In the present study, whole black rice was added directly to the mouse diets, so the amounts of anthocyanins in the mouse diets were limited. However, in addition to anthocyanins, black rice also contains other flavonoids, terpenoids, and some beneficial proteins and polysaccharides (Dias et al., 2017), which may have some beneficial effects on the body.

Shen et al. (2017) reported that adding 51% of sorghum flour to the diet had significant beneficial effects on TG, TC, HDL‐C, and LDL‐C. However, Moraes et al. (2018) found that the addition of 19% whole grain sorghum flour or decorticated sorghum flour or 12% sorghum bran to the diet did not significantly affect the TG, TC, HDL‐C, or LDL‐C levels in obese mice. The results of the present study showed that sorghum rice had an effect similar to that of black rice. The antioxidants in sorghum rice probably played a role in relieving oxidative stress in the hypercholesterolemic mice, but they did not reach the effect shown by Shen et al. This may be related to the use of fully decorticated sorghum rice, rather than whole sorghum rice, in the present study, or to the type of animal model used.

### 3.3 Effects of black rice and sorghum rice diets on the morphology of mice liver

Hepatic steatosis is a typical nonalcoholic fatty liver disease associated with hyperlipidemia and obesity (Zheng et al., 2008). High cholesterol is also associated with reduced anti‐inflammatory ability, which may contribute to hepatocyte death (Yeap et al., 2015).

**Figure 2:**
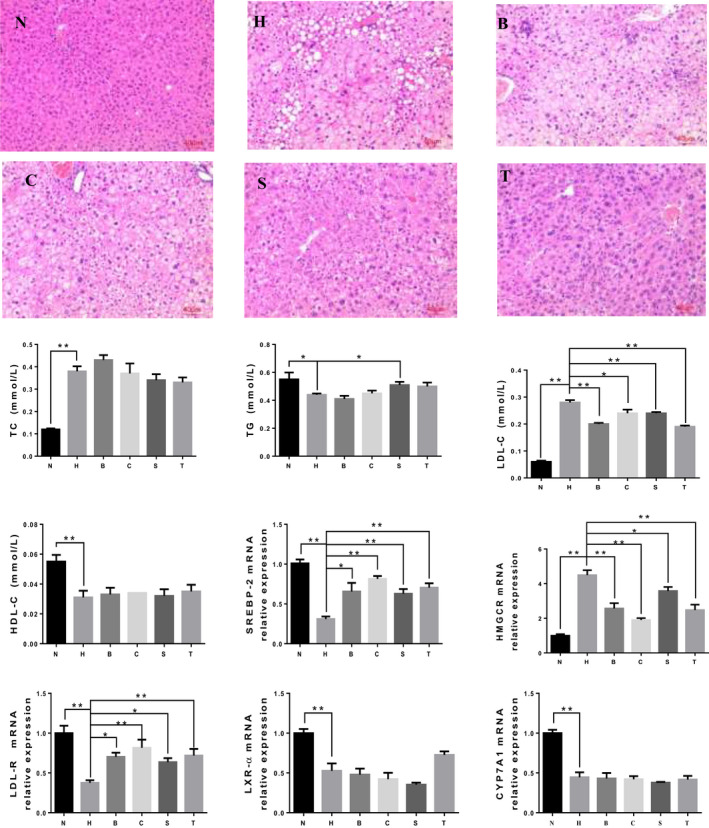
Effects of diets containing black rice or sorghum rice on histologically sliced of mice liver under light magnification, and expression of genes involved in cholesterol metabolism in the mouse liver. Histologically data shown are representative images (magnification 100×). Other data are presented as means ± *SD*. Means with *, ** were significantly different (*p* < .05, *p* < .01) compared with group H, without * means no difference (*p* > .05).Typical photographs of liver sections of N: normal group; H: high cholesterol diet group; B: low dose black rice group; C: high dose black rice group; S: low dose sorghum rice group; T: high dose sorghum rice group

Figure 2 shows that no lipid droplets formed in the mouse liver cells of the group N; the liver cells were structurally intact. However, the mouse liver cells in the group H were disordered, with large lipid droplets were apparent in the cytoplasm. The numbers of large lipid droplets in the mouse liver cells of B, C, S, and T were reduced, although small lipid droplets were still apparent and some cell nuclei are not centered, indicating that the pathological changes in the liver tissue were improved compared with those in H.

### 3.4 Effects of black rice and sorghum rice diets on liver lipids in mice

The results of the liver lipid analysis showed that the liver TC levels in H were significantly higher than those in *N* (Figure 2). Diets containing different proportions of sorghum or black rice had no effects on the liver TC, TG, and HDL‐C levels in the hypercholesterolemic mice. Zawistowski et al. (2009) demonstrated that the high‐purity anthocyanins extracted from black rice reduced the TC levels in the livers of hypercholesterolemic mice. Black rice added to the diets in the present study had a limited effect on the blood lipid metabolism parameters of hypercholesterolemic mice. However, a very notable result was that Sorghum rice diets and black rice diet had significantly reduced effects on the liver LDL‐C levels in these mice (*p* < .05).

### 3.5 Effects of black rice and sorghum rice diets on the expression of genes involved in cholesterol metabolism in the mice liver

The expression of liver SREBP‐2 mRNA was significantly lower in the group H than in the group *N* (Figure 2). The expression of liver SREBP‐2 mRNA was significantly higher in all the black rice and sorghum rice groups than in the group H, and the increases in the high‐dose groups (C,T) were greater than those in the low‐dose groups. The expression of liver HMG‐CoA reductase mRNA was significantly higher in the group H than in the group N. The expression of liver HMG‐CoA reductase mRNA was significantly lower in all the black rice and sorghum rice groups than in the group H, and the reductions in the high‐dose groups were greater than those in the low‐dose groups.

SREBP‐2 specifically regulates the cholesterol and fatty acid metabolism (Joseph et al., 2002), and its most important target gene encodes HMG‐CoA reductase, the rate‐limiting enzyme in the cholesterol synthesis pathway (Reihner et al., 1990). Statins are cholesterol‐lowering drugs, and their main mechanism of action is in inhibiting HMG‐CoA reductase activity. The experimental results in this study showed that both the black rice and sorghum rice diets reduced the synthesis of liver cholesterol and that the diets containing higher proportions had better effects.

The expression of liver LDL‐R mRNA was significantly lower in the group H than in the group N. The expression of liver LDL‐R mRNA was significantly higher in all the black rice and sorghum rice groups than in H. LDL‐R plays a dual role in LDL metabolism (Hill et al., 2003): LDL‐R limits the synthesis of LDL by reducing LDL density lipoprotein (IDL) circulating in the blood. The downregulation of this cholesterol‐clearing receptor resulted in lower long‐term cholesterol levels in the liver cells than in the extracellular space, which could not meet the metabolic needs of the body. As a result, the HMG‐CoA cholesterol biosynthetic pathway was activated, leading to increased blood cholesterol levels in the group H (Zhang, 2017). Our experimental results showed that both the black rice and sorghum rice diets increased the levels of LDL‐R in the mice, thereby increasing their intracellular cholesterol concentrations and lowering their blood cholesterol concentrations. As shown in Figure 2, the LDL‐C levels in some proportions of black rice and sorghum rice groups were lower than those in group H; it was consistent with the increase of expression of liver LDL‐R mRNA.

The expression of liver LXR‐ɑ mRNA was significantly lower in the group H than in the group N. The expression of liver LXR‐ɑ mRNA was significantly lower in all the black rice groups than in the group H, whereas its expression was only reduced in the low‐dose sorghum rice group. The results suggested that the mice liver cholesterol accumulation is overmuch due to high‐cholesterol diet, and the absorption of cholesterol in the liver was inhibited (Reyesquiroz et al., 2014). The expression of the *CPY71A* gene was significantly lower in the group H than in the group N. The expressions of liver CPY71A mRNA were not significantly different between B, C, S, T, and H group. LXR‐ɑ is an important nuclear receptor in lipid metabolism, controlling the conversion of cholesterol to bile acids, mainly by regulating the mRNA expression of CPY71A, a rate‐limiting enzyme of the bile acid synthesis pathway in the liver (Mitchell et al., 1991).

Our experimental results showed that the synthesis of cholesterol in the liver was inhibited through HMG‐CoA reductase and SREBP‐2 pathway, and cholesterol excretion increased through LDL‐R pathway by eating black rice and sorghum rice for a long time.

### 3.6 Effects of black rice and sorghum rice diets on intestinal villus structure

Villous morphology reflects the function of intestinal absorption. High and dense intestinal villi increase the intestinal absorption area and can fully absorb nutrients from food. On the contrary, damaged intestinal villi and villous loss will weaken digestion and absorption.

**Figure 3:**
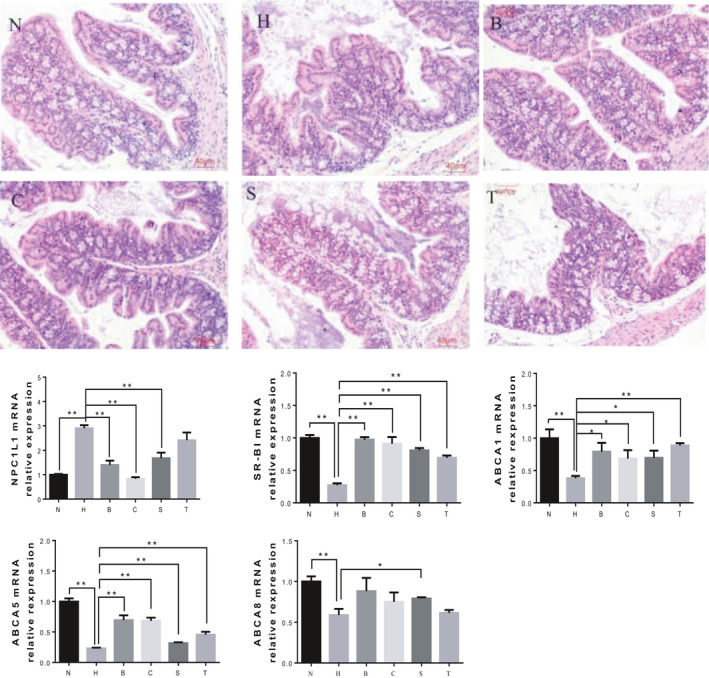
Effects of diets containing black rice or sorghum rice on intestinal tissue morphology and expression of genes involved in cholesterol metabolism in the mice intestines of hypercholesterolemic mice. Histologically data shown are representative images (magnification 100×). Other data are presented as means ± *SD*. Means with *, ** were significantly different (*p* < .05, *p* < .01) compared with group H, without * means no difference (*p* > .05). N: normal group; H: high cholesterol diet group; B: low dose black rice group; C: high dose black rice group; S: low dose sorghum rice group; T: high dose sorghum rice group

It can be observed in Figure 3 that mouse small intestinal villi and intestinal mucosa in N were integral. The small intestinal villi in N were high and densely arranged. In contrast, the height of small intestinal villi in H was reduced, some villi were absent, the lamina propria was swollen, and the lamina propria was separated from the epithelial layer, inflammatory cells infiltrated in the mucosal layer, and the mucosal muscular layer was edema, indicating that the small intestinal villi and intestinal mucosa in H were damaged. The microscopic observations of the small intestine sections in the low‐ and high‐dose black rice groups showed that the integrity and arrangement of the intestinal villi were improved compared with H. Those data indicate that the damaged small intestine villi had recovered to some extent. Although the small intestinal villi were sparse in some places, the intestinal mucosa was integral. On the contrary, the villi and mucosa of the small intestine were not repaired in sorghum groups, and the mucosal muscularis was still swelling seriously.

### 3.7 Effects of black rice and sorghum rice diets on the expression of genes related to mouse intestinal cholesterol metabolism

The expression of NPC1L1 mRNA in the intestinal tract of group H was significantly increased relative to group *N* (Figure 3). Compared with H, the expression of NPC1L1 mRNA in the mouse intestine of two black rice groups was significantly decreased. However, in the sorghum rice groups, only low‐dose sorghum rice significantly reduced the expression of NPC1L1 mRNA in the mouse intestine. NPC1L1 protein expressed in the brush border of intestinal epithelial cells is an important protein involved in cholesterol absorption in the small intestine (Jr & Altmann, 2009). It mainly mediates the absorption of free cholesterol from the diet and bile into the intestinal mucosa. When the expression level of NPC1L1 mRNA is decreased, the cholesterol absorbed from the intestinal tract is reduced. The experimental results showed that diets containing different proportions of black rice and a low proportion of sorghum rice reduced intestinal cholesterol absorption.

The expression of SR‐B1 mRNA was significantly lower in H than in *N* (Figure 3). The expression of liver SR‐B1 mRNA was significantly higher in all the black rice and sorghum rice groups than in H. The mechanism of hypercholesterolemia may be related to the low expression of LDL‐R (LDL receptor) and SR‐B1. Scavenger receptor type B type I (SR‐BI) is a receptor protein which is currently recognized the only HDL receptor (Kozarsky et al., 1997). After entering the intestine, free cholesterol will bind to HDL under the mediation of SR‐BI and form HDL‐C, which will be transported back to the liver for further decomposition. Black rice and sorghum rice diets both increased the expression of SR‐BI mRNA, thus improving the reverse cholesterol transport.

The expression of ABCA1 mRNA was significantly decreased in H relative to *N* (Figure 3). Compared with H, the expression levels of ABCA1 mRNA in the mouse liver were increased significantly in black rice and sorghum rice groups. ABCA1 is a key protein in the formation of HDL (Wang et al., 2001). The primary role of ABCA1 is to transport intracellular cholesterol and phospholipids to the cell surface to form HDL, which is an acceptor for ABCG1‐mediated cholesterol efflux. The results showed that a diet containing all proportions of black rice and sorghum rice promoted the expression of ABCA1 mRNA and increased the expression level of HDL mRNA.

The expression of ABCG5 and ABCG8 mRNA in the intestinal tract was significantly decreased in H, compared to N. Compared with H, the expression levels of ABCG5 mRNA in the mouse liver were significantly increased in all the black rice groups and sorghum rice groups. In addition, compared with H, the expression levels of ABCG8 mRNA were slightly increased in B, C, T, significantly increased in group S. ABCG5/ABCG8 proteins are co‐expressed in the brush border of intestinal epithelial cells and work together as a dimer, which reversely transports free cholesterol absorbed in the small intestine into the intestine lumen for fecal excretion (Wang et al., 2015). Studies have shown that ABCG8 mRNA deficiency in mice significantly increased small intestinal cholesterol absorption, while overexpression of ABCG5 and ABCG8 mRNA in mice reduced cholesterol absorption (Yu et al., 2014). The experimental results showed that diets containing black rice and sorghum rice, especially low dose of sorghum rice, increased the expression levels of ABCG5/ABCG8 mRNA in the small intestine, which reduced the cholesterol absorption in the small intestine.

### 3.8 Effects of black rice and sorghum rice diets on microbiota diversity in the intestinal feces of hypercholesterolemic mice

**Figure 4:**
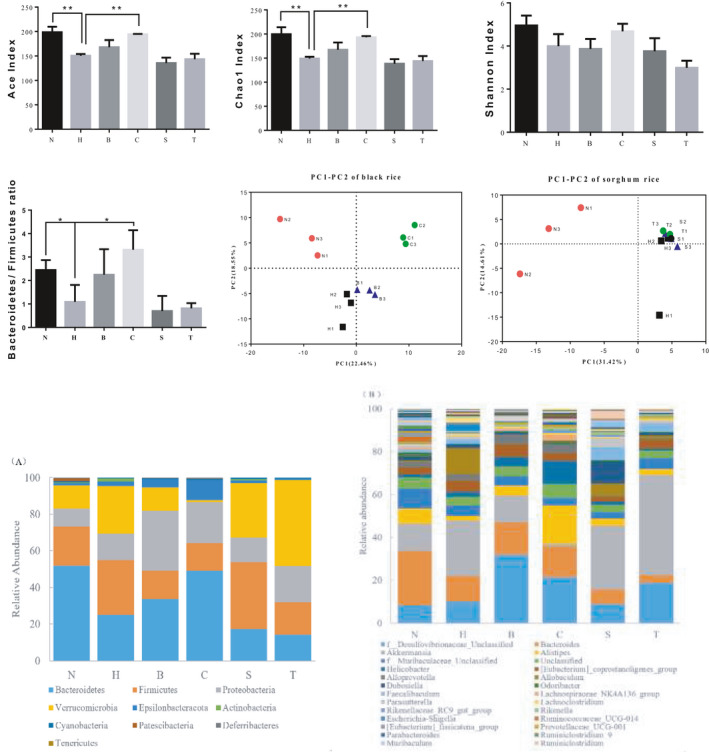
Effects of black rice and sorghum rice diets on microbiota diversity, Principal Component Analysis (PCA) of microbial communities, and ratio of *Bacteroidetes* to *Firmicutes*, intestinal microbiota at the phylum level (a) and the genus level (b). The data are presented as means ± *SD*. Means with *, ** were significantly different (*p* < .05, *p* < .01) compared with group H, without * means no difference (*p* > .05). N: normal group; H: high cholesterol diet group; B: low dose black rice group; C: high dose black rice group; S: low dose sorghum rice group; T: high dose sorghum rice group

The alpha diversity indices were used for comparison. Common alpha diversity indices include ACE, Chao1, Shannon. ACE and Chao1 indices predict the abundance of microorganisms in the sample based on the measured OTU—the greater the values of the indices, the higher the species richness. Shannon indices reflect the number of species in the intestinal microbiota and the proportion of each species in the population. The greater the Shannon value, the higher the species richness. The results in Figure 4 show that compared with N, the number of microbial species and the proportion of each species in the intestinal feces in H decreased significantly. This result was consistent with the studies of Wang (Wang et al., 2018). Compared with H, microbial diversity indexes in the intestinal feces in C were significantly increased, while the microbial abundance in B and the two sorghum rice groups were not significantly changed. However, compared with group H, the microbial species of sorghum groups showed a decreasing trend. The results indicate that high proportion of black rice diet can effectively repair the intestinal microbiota ecosystem damaged by high‐cholesterol diet, but there were no similar benefits from eating sorghum.

Beta diversity was calculated, and principal component analysis (PCA) plots were drawn to compare microbial communities from different intestinal feces samples, respectively. It can be observed in Figure 4; intestinal microbiota from N and H demonstrated higher divergence and were separated clearly by PCA. In addition, intestinal microbiota from H and C were separated clearly. In contrast, samples from H, S, and T displayed closer relative distances and were difficult to distinguish (Figure 4). These results indicated that the intestinal microbial communities in H became obviously different from those in N after a high‐cholesterol diet. The intestinal microbiota damaged from high‐cholesterol diet could be improved significantly by high‐dose black rice diet, while sorghum diets had little effect on the damage. The reason might be that sorghum contains plenty of tannins, which reduce intestinal digestibility to sorghum and might inhibit the growth of some intestinal flora (Sarwar et al., 2012).

Figure 4 illustrates the effects of black rice and sorghum rice diets on intestinal microbiota damaged from a high‐cholesterol diet. Figure 4a shows that the four most abundant phyla in high‐cholesterol diet‐induced mice included *Bacteroidetes, Firmicutes, Proteobacteria, and Verrucomicrobia*. The total relative abundance of these four phyla was over 95% in all groups. The relative abundance of *Bacteroidetes* in N was the highest, reaching 50%. There was a big difference in intestinal microbiota between H and N. *Firmicutes* was dominant in the intestinal microbiota in H, and the relative abundance of *Bacteroidetes* was significantly reduced (*p* < .05) to about 25%. The composition of intestinal microbiota in S and T were similar to those in H. In addition, the relative abundances of *Verrucomicrobia* increased obviously in S (30%) and T (47%) compared to H, whereas those of *Bacteroidetes* decreased significantly (17% in S and 12% in T) accompanied by the increase of sorghum rice proportion in diet. However, compared to H, the relative abundances of *Bacteroidetes* in two black rice groups were increased (30% in B and 50% in C) substantially and were higher than that of *Firmicutes*.

At taxonomic levels, more differences in relative abundances were observed. Figure 4b shows that *Desulfovibrionaceae, Bacteroides, Akkermansia,* and *Alistipes* were the main dominant flora in N, with a total relative abundance of 53%. Compared with N, the relative abundances of *Bacteroides* and *Alistipes* in H decreased significantly, whereas that of Akkermansia increased obviously. Compared with H, the relative abundances of *Desulfovibrionaceae*, *Bacteroides and Alistipes* increased and those of *Akkermansia* decreased in the intestinal microbiota in B and C. On the contrary, the relative abundances of *Akkermansia* increased, but that of *Bacteroides* decreased in the intestinal microbiota in S and T.

Ley et al. (2006) believed that the relative proportion of *Bacteroidetes* was decreased in obese people by comparison with lean people. Turnbaugh et al. (2006) thought that obesity was associated with changes in the relative abundance of the two intestinal dominant bacterial divisions, the *Bacteroidetes* and the *Firmicutes*. It could be observed in Figure 4 that a lower ratio of B/*F* (*Bacteroidetes/ Firmicutes*) was found in group H. It indicates that a high‐cholesterol diet can induce a lower ratio of B/F, just like a high‐fat diet (Huang et al., 2019). As reported by Turnbaugh et al. (2006), low ratio of B/F had a negative impact on health because it may lead to obesity. High dose of black rice diet promoted the health of the intestinal microbiota for its higher ratio of B/F, but high dose of sorghum diet continued to reduce the ratio of B/F after a high‐cholesterol diet.

## 4 CONCLUSION

Our hypothesis was proved in this study that different doses of sorghum rice and black rice produced different effects on cholesterol metabolism and intestinal microbiota. The effects of black rice and sorghum rice on the lipid metabolism in hypercholesterolemic mice were investigated in this study. Our results showed that at the genetic level, diets containing different proportions of black rice or sorghum rice had certain beneficial effects on cholesterol synthesis in the liver after the mice were fed the diets for 12 weeks. However, at the biochemical level, the diets containing different proportions of black rice or sorghum rice had no significant effect on serum and liver TC in these mice, but they significantly reduced serum TG and liver LDL‐C levels after the mice were fed the diets for 12 weeks.

Diets containing different proportions of black rice can reduced the absorption of cholesterol and promoting the reverse transportation of cholesterol in the intestine by regulating the expression levels of relative genes. In addition, black rice diets can also repair intestinal tissues damaged from a high‐cholesterol diet and increase the proportions of beneficial intestinal bacteria. Although sorghum diets can also reduce the absorption of cholesterol in intestine, they have no beneficial effects on the intestinal mucosal structure and intestinal bacteria. Therefore, eating black rice seems more beneficial to cholesterol metabolism and intestinal flora stability than eating sorghum through overall consideration.

## CONFLICT OF INTEREST

There are no conflicts of interest to declare.

## AUTHOR CONTRIBUTION

Haiying Liu involved in conceptualization, methodology, project administration, resources, writing—review and editing, and funding acquisition. Lu Huang involved in software and writing—original draft. Xinli Pei involved in data curation, software, and formal analysis.

## Supporting information

Appendix S1

## Data Availability

The data that support the findings of this study are openly available in [repository name, e.g. “figshare”] at xxxx, reference number [reference number].
