# Supplementary material for: Effects of sorghum rice and black rice on genes associated with cholesterol metabolism in hypercholesterolemic mice liver and intestine
Source: Food Sci Nutr. 2020 Nov 14;9(1):217–29. doi: 10.1002/fsn3.1986 (PMC7802551; doi:10.1002/fsn3.1986)
Supplement: Supplementary file 1 — Appendix S1 [file FSN3-9-217-s001.docx]

**Supplementary material**

**Effects of sorghum rice and black rice on genes associated with cholesterol metabolism in hypercholesterolemic mice liver and intestine**

Haiying Liu ^a,b*^, Lu Huang ^b^ , Xinli Pei ^b^

^a^State Key Laboratory of Food Science and Technology, Jiangnan University, Wuxi, Jiangsu, People’s Republic of China;

^b^School of Food Science and Technology, Jiangnan University, Wuxi, Jiangsu, People’s Republic of China;

*Corresponding author: Haiying. Liu

Tel.: +86-510-85329076; Fax: +86-510-85329076.

E-mail address: liuhaiying@jiangnan.edu.cn (Haiying Liu)

Table S1 The basic components of Black rice and sorghum (w/w, %)

| Basic components | | | | |
| --- | --- | --- | --- | --- |
|  | Moisture | Protein | Oil | Starch |
| Black rice | 10.80±0.11 | 9.82±0.52 | 2.32±0.17 | 70.82±0.31 |
| Sorghum | 14.23±0.05 | 10.42±0.39 | 3.12±0.05 | 66.32±0.55 |

|  |  |
| --- | --- |
|  |  |

Fig.S1 Effects of diets containing low or high proportions of black rice or sorghum on liver lipids and the expression of genes involved in cholesterol metabolism in the mouse liver. The data are presented as means ± SD. Means with *, ** were significantly different (P<0.05, P<0.01) compared with group H, without * means no difference (P>0.05). N: normal group; H: high cholesterol diet group; B: low dose black rice group; C: high dose black rice group; S: low dose sorghum group; T: high dose sorghum group.

Hmox1
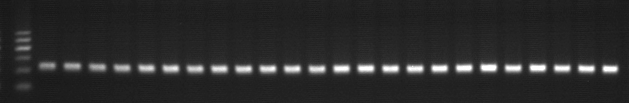

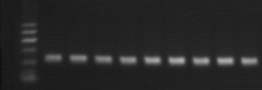


Abcg8
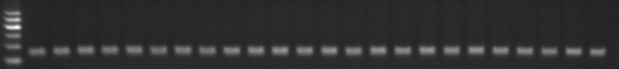

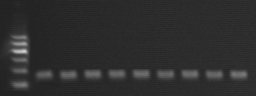


Scarb1
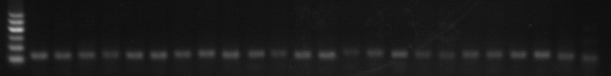

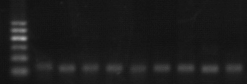


Abcg5
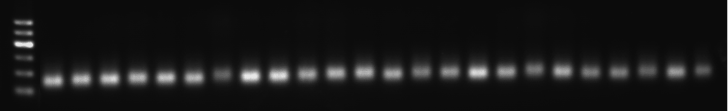

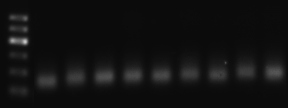


ABC1
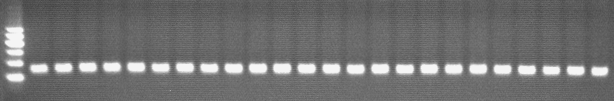

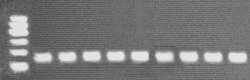


Nrf2
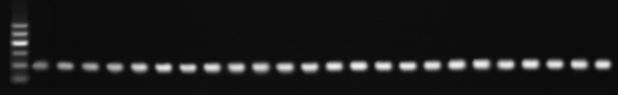

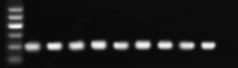


Cyp7a1
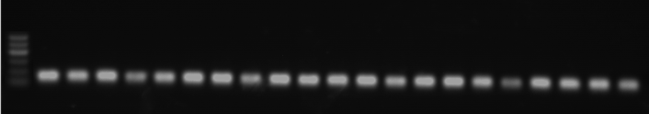

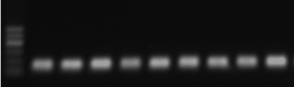


LXR-a
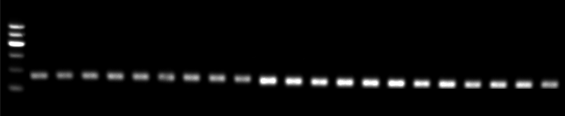

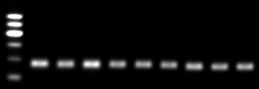


Npc1l1
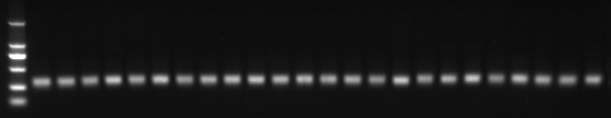

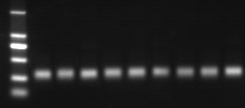


Cat
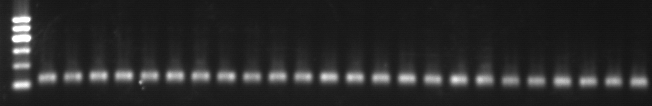

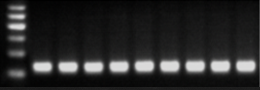


Hmgcr
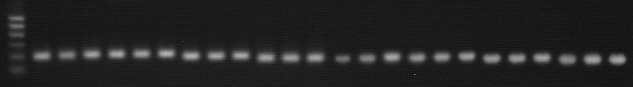

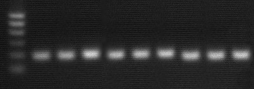


Sod1
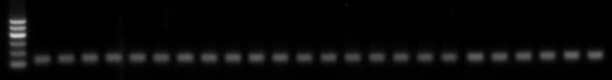

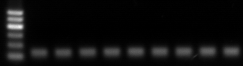


Srebf2
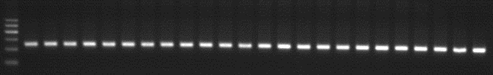

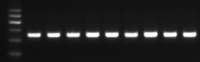


LDL-R
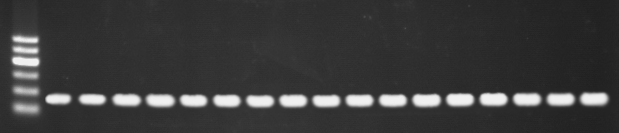

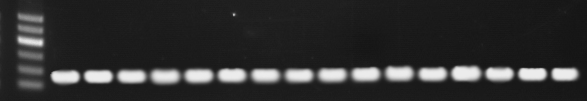


Fig.S2 Electrophoretogram of PCR amplified 18S rRNA of the associated gene.

Sample order from left to right is N1，N2，N3，H1，H2，H3，B1，B2，B3，C1，C2，C3，O1，O2，O3，P1，P2，P3，S1，S2，S3，T1，T2，T3，F1，F2，F3，G1，G2，G3，R1，R2，R3. N: normal group; H: high cholesterol diet group; B: low dose black rice group; C: high dose black rice group; S: low dose sorghum group; T: high dose sorghum group.


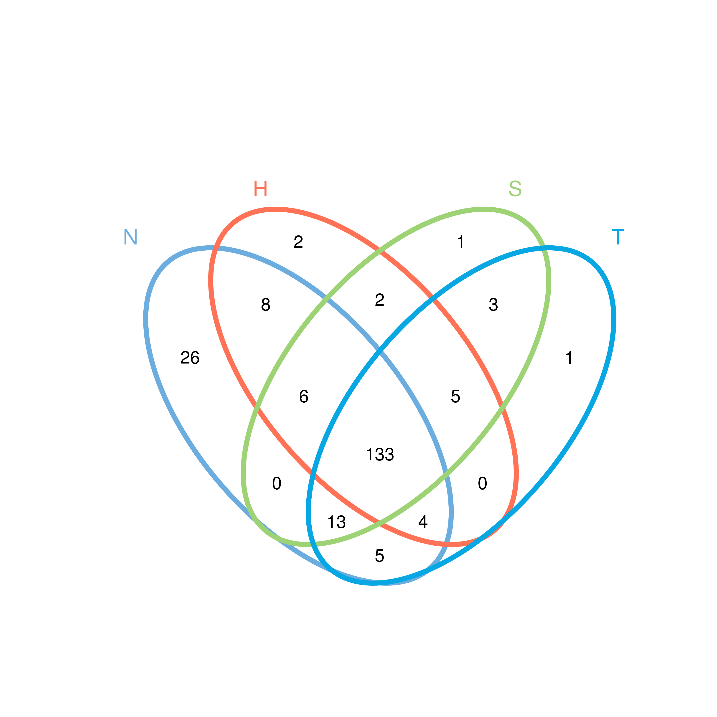

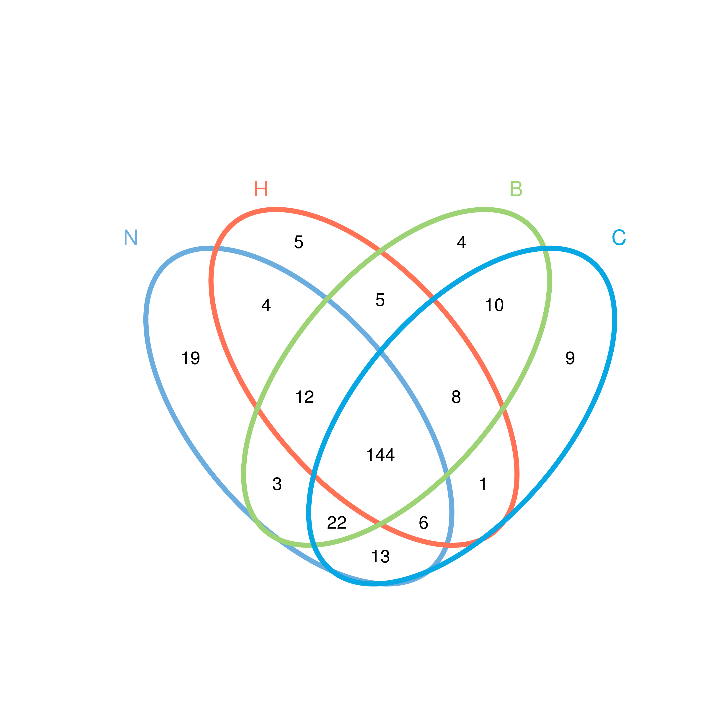


Fig.S3 Venn diagrams of microbiota in the small intestines of mice.N: normal group; H: high cholesterol diet group; B: low dose black rice group; C: high dose black rice group; S: low dose sorghum group; T: high dose sorghum group.


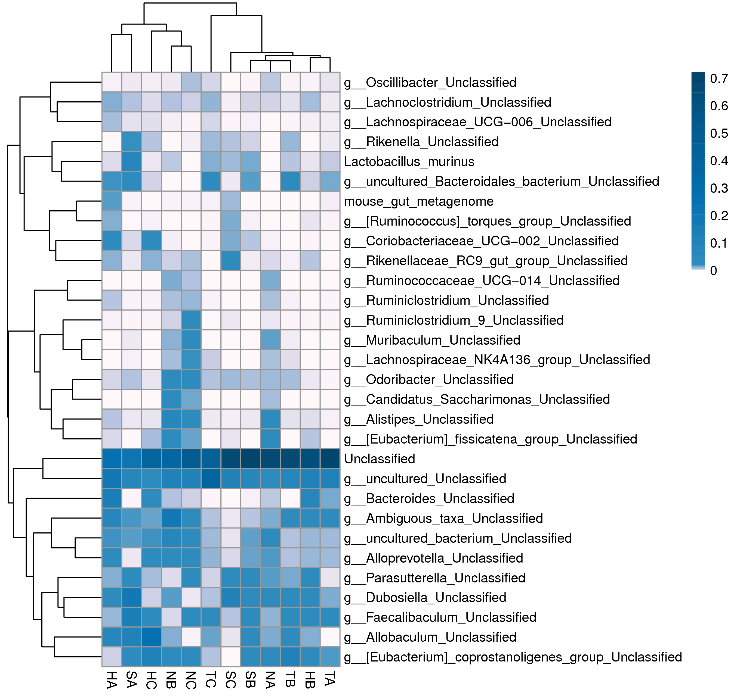

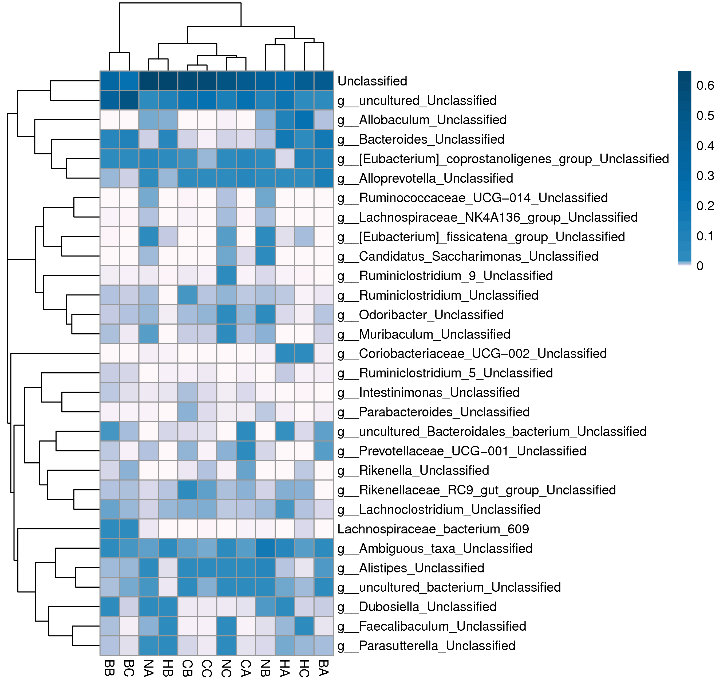


Fig.S4 Heat map of species distribution of microbiota in the small intestines of mice. N: normal group; H: high cholesterol diet group; B: low dose black rice group; C: high dose black rice group; S: low dose sorghum group; T: high dose sorghum group.
